# Explainable AI in medicine: challenges of integrating XAI into the future clinical routine

**DOI:** 10.3389/fradi.2025.1627169

**Published:** 2025-08-05

**Authors:** Tim Räz, Aurélie Pahud De Mortanges, Mauricio Reyes

**Affiliations:** ^1^ Institute of Philosophy, University of Bern, Bern, Switzerland; ^2^ ARTORG Center for Biomedical Research, University of Bern, Bern, Switzerland; ^3^ Department of Radiation Oncology, University Hospital Bern, University of Bern, Bern, Switzerland

**Keywords:** explainable AI, interpretability, multimodal AI, clinical applications, philosophy of AI

## Abstract

Future AI systems may need to provide medical professionals with explanations of AI predictions and decisions. While current XAI methods match these requirements in principle, they are too inflexible and not sufficiently geared toward clinicians’ needs to fulfill this role. This paper offers a conceptual roadmap for how XAI may be integrated into future medical practice. We identify three desiderata of increasing difficulty: First, explanations need to be provided in a context- and user-dependent manner. Second, explanations need to be created through a genuine dialogue between AI and human users. Third, AI systems need genuine social capabilities. We use an imaginary stroke treatment scenario as a foundation for our roadmap to explore how the three challenges emerge at different stages of clinical practice. We provide definitions of key concepts such as genuine dialogue and social capability, we discuss why these capabilities are desirable, and we identify major roadblocks. Our goal is to help practitioners and researchers in developing future XAI that is capable of operating as a participant in complex medical environments. We employ an interdisciplinary methodology that integrates medical XAI, medical practice, and philosophy.

## 1 Introduction

**Figure 1:**
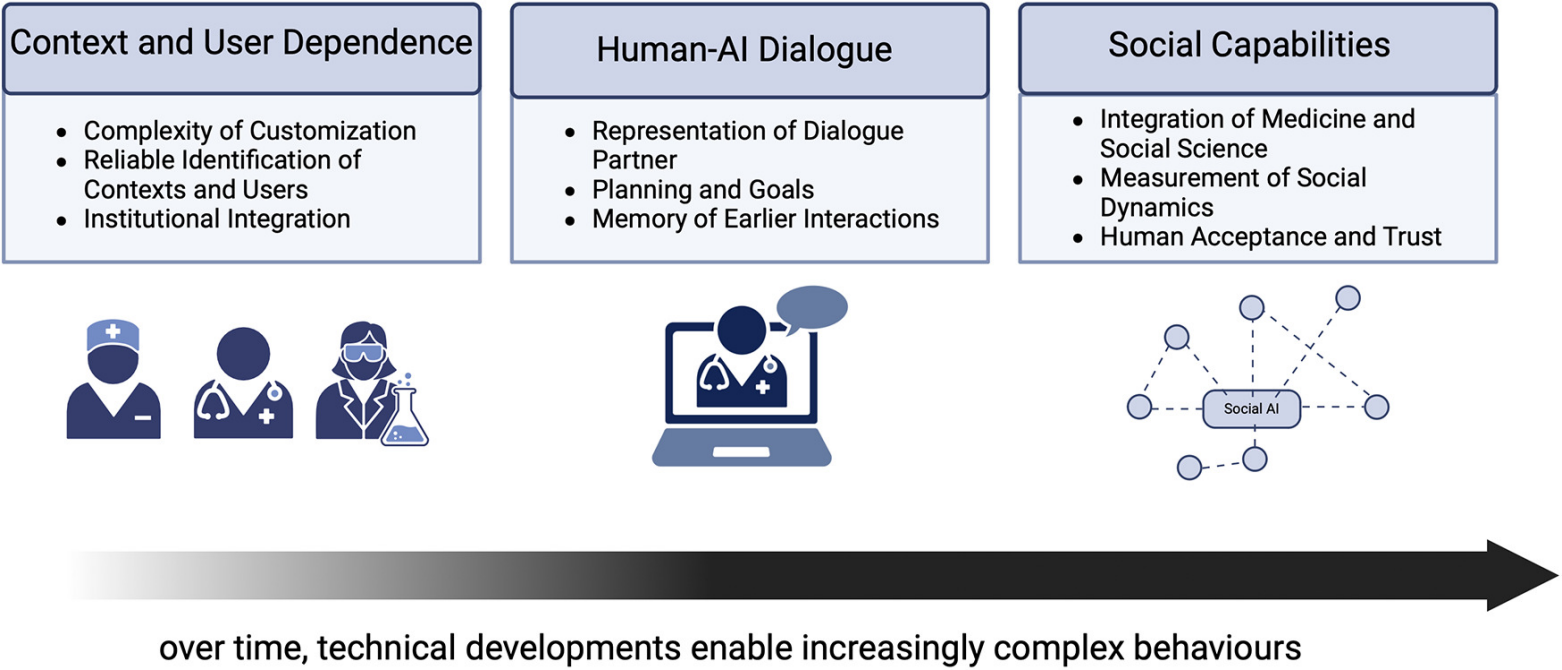
Graphical abstract and summary of contents. Over time, technical developments will likely enable XAI systems to complete increasingly complex tasks. While context- and user dependence might be addressed in the near future, human-AI dialogue and social capabilities might only arise in a more distant future.

Currently, AI in medicine is a tool among many, akin to an imaging or measuring device. The black-box nature of AI has motivated research in explainable AI (XAI), a subfield of AI. XAI deals with the problem of providing insights as to how an AI system uses information to solve a given task (1, 2). In this paradigm, incorporating human-in-the-loop aspects in AI-based systems is an important development (3). In the future, medical AI may take a more active role, interactions between AI systems and medical professionals may become more complex, and AI may become a full participant in the medical workspace. To do so, future AI systems will need to provide medical professionals with insight into their reasoning process, explaining and justifying their contributions. In principle, XAI methods match these requirements, because XAI is designed to provide insight into predictions and decisions by AI systems. However, current XAI methods are too inflexible and not sufficiently geared toward clinicians’ needs to fit the bill for future AI (4). The present paper discusses how this integration of AI via XAI may be achieved. We identify three desiderata of increasing difficulty that must be met before AI is fully integrated into medical practice through XAI. First, explanations need to be provided in a context- and user-dependent manner. Second, explanations need to be created through a genuine dialogue between AI and human users; we provide three criteria for genuine dialogue. Third, AI systems need genuine social capabilities, to be explicitly defined below; see Figure 1 for an overview of the three desiderata. We discuss why these abilities would be desirable and we identify major roadblocks. We employ an interdisciplinary methodology, integrating expertise from medical XAI, medical practice, and philosophy. The first two provide a firm footing in the practice of XAI and medicine, while the philosophical perspective highlights the potential and the challenges of XAI. Based on current literature, the paper offers a philosophically grounded conceptual roadmap to the future of XAI in medical practice.

We first provide background and discuss related work from medical XAI and philosophy (Section 2). We then present a use case, an imaginary stroke scenario outlining a standard medical treatment cycle. The scenario serves as the backdrop for our discussion of the future of XAI in medical practice (Section 3). Then we turn to the discussion of how XAI may be integrated into the different phases of the use case, grouped into the three challenges of context- and user dependence, human-AI dialogue, and social capabilities (Section 4).

## 2 Background, related work

The current state of the art in Explainable AI (XAI) for medicine mainly revolves around methodologies originally developed in the fields of computer vision and related domains, which have since been adopted and/or adapted to clinical information, such as imaging, text- and tabular-based patient clinical information. Beyond methodological adaptations, a growing number of experimental studies in radiology and medical image analysis have begun to apply XAI techniques in practice and assess their interpretability with medical experts. For instance, some studies combine saliency-based heatmaps or feature attributions with clinical decision tasks, and validate their plausibility through qualitative feedback from radiologists or domain specialists. These investigations remain largely at the research or prototyping stage, but they demonstrate the feasibility and relevance of XAI in imaging workflows, and provide valuable insights into how clinicians interact with and assess such tools (1, 2, 5–10).

Explainable Artificial Intelligence (XAI) has emerged as a critical research area, addressing the growing need for transparency in deep learning models across various domains. Recent surveys categorize XAI methods into post-hoc interpretability techniques, such as saliency maps, attention mechanisms, and feature-attribution models, and intrinsically interpretable architectures that incorporate explainability directly into model design (1, 2). Among these, saliency-based methods, such as Grad-CAM, Layer-wise Relevance Propagation (LRP), and Integrated Gradients are widely used to visualize the features most influential to a model’s prediction.

Beyond post-hoc visualization, recent work has explored how XAI can actively guide model training, ensuring that models learn meaningful patterns rather than spurious correlations. Mahapatra et al. (11) introduced an interpretability-guided inductive bias that enforces spatial coherence in saliency maps and encourages class-distinctiveness during training. By integrating interpretability constraints into the learning process, their approach improves both predictive performance and the reliability of generated explanations.

A key challenge in deploying XAI across different fields is shortcut learning (12), where models exploit spurious correlations in the data rather than genuinely informative features. Saliency-based methods have been instrumental in uncovering such issues. For example, attention-based saliency maps have been used to reveal cases where models relied on unintended artifacts in the data, leading to incorrect predictions (13). These findings highlight how XAI techniques can expose and help mitigate hidden biases in model behavior.

Even as XAI methods enhance transparency, their real-world utility remains an open question. Ihongbe et al. (14) conducted a user-centered study evaluating interpretability methods such as Grad-CAM and LIME. While participants found Grad-CAM generally more intuitive and reliable, the study revealed a broader issue: limited awareness among practitioners regarding the practical value of explainability tools. This underlines the need for structured evaluation frameworks to assess whether XAI genuinely improves decision-making processes rather than simply producing visually plausible outputs.

XAI has also played a vital role in detecting hidden biases. Gichoya et al. (15) demonstrated that deep learning models could infer sensitive attributes, such as patient race, from imaging data, even when such information was not explicitly provided. Using saliency maps and ablation studies, they showed how models leveraged subtle features correlated with race, raising important ethical and operational concerns regarding AI deployment in sensitive contexts.

Multiple XAI toolboxes with different functionalities have been developed, such as Captum (16), Quantus (17), and Alibi Explain (18). While some of these toolboxes can handle diverse data types, many current XAI implementations remain mono-modal, focusing on a single data modality. This is a significant limitation, as real-world decision-making often involves integrating multimodal information–such as combining visual, textual, and tabular data. Recent surveys, such as (19), outline a diverse range of multimodal XAI approaches, especially in non-medical domains like multimedia reasoning and robotics, where explanatory outputs may combine visual, textual, and symbolic content. In contrast, most clinical XAI systems remain mono-explanatory: even when built on multimodal inputs (e.g., imaging, labs, and clinical text), explanations are typically produced per modality in isolation. A notable conceptual contribution comes from (20), who propose a framework for multimodal explainability for medical imaging that uses different types of XAI outputs (e.g, saliency-based visual outputs with structured clinical data explanations) to enable contrastive, context-aware explanations. While their work does not implement a full system, it highlights the need for XAI designs in which the explanation itself, not just the model input, is truly multimodal. This remains a key open direction in clinical AI.

Furthermore, most current XAI systems are designed with a developer-centric perspective rather than being tailored to the needs and workflows of domain experts and end-users (21, 22). Additionally, the vast majority of studies still focus on isolated time points, while many real-world scenarios depend on longitudinal information. The limitations of current XAI methods are well recognized (23), and there is ongoing debate about whether XAI techniques or inherently interpretable models may offer more robust solutions to overcoming AI opacity (24, 25).

Conceptual requirements of explanations for XAI have been discussed in philosophy (26–28) as well as in the technical literature mentioned above. Explanations of AI output are constrained by two desiderata. First, an explanation should provide accurate and relevant information about how the output came about. If explanatory information is not accurate or relevant, it does not achieve its goal. Second, the explanatory information has to be provided in an understandable manner. From a practical implementation point of view, these criteria can be translated into concrete design goals. Accuracy requires that explanations reflect the model’s actual reasoning process, not post-hoc rationalizations. Relevance implies tailoring content to the user’s clinical role and context, prioritizing what matters most for decision-making. Understandability calls for presenting explanations in familiar formats (e.g., visual overlays or structured text) and at an appropriate level of detail, ideally adjustable based on user preference or task demands.

Even accurate and relevant information fails to explain if it cannot be grasped (29). Due to the time constraints in clinical settings, XAI must be precise and concise. One of the main challenges of developing XAI is that these two desiderata are in tension and must be weighed against each other. Whether explanatory information can be grasped depends on several factors (30). First, context determines the purpose of an explanation, which in turn influences whether an explanation is adequate. For example, understanding possible causes of symptoms looks different in an emergency and in a research situation (31). Second, to be understandable, explanations may need to be customized for different users. For example, a research physician has different explanatory needs than a nurse in an ICU (32). Below, we will expand on context- and user-dependence.

## 3 Use case: stroke scenario

**Figure 2:**
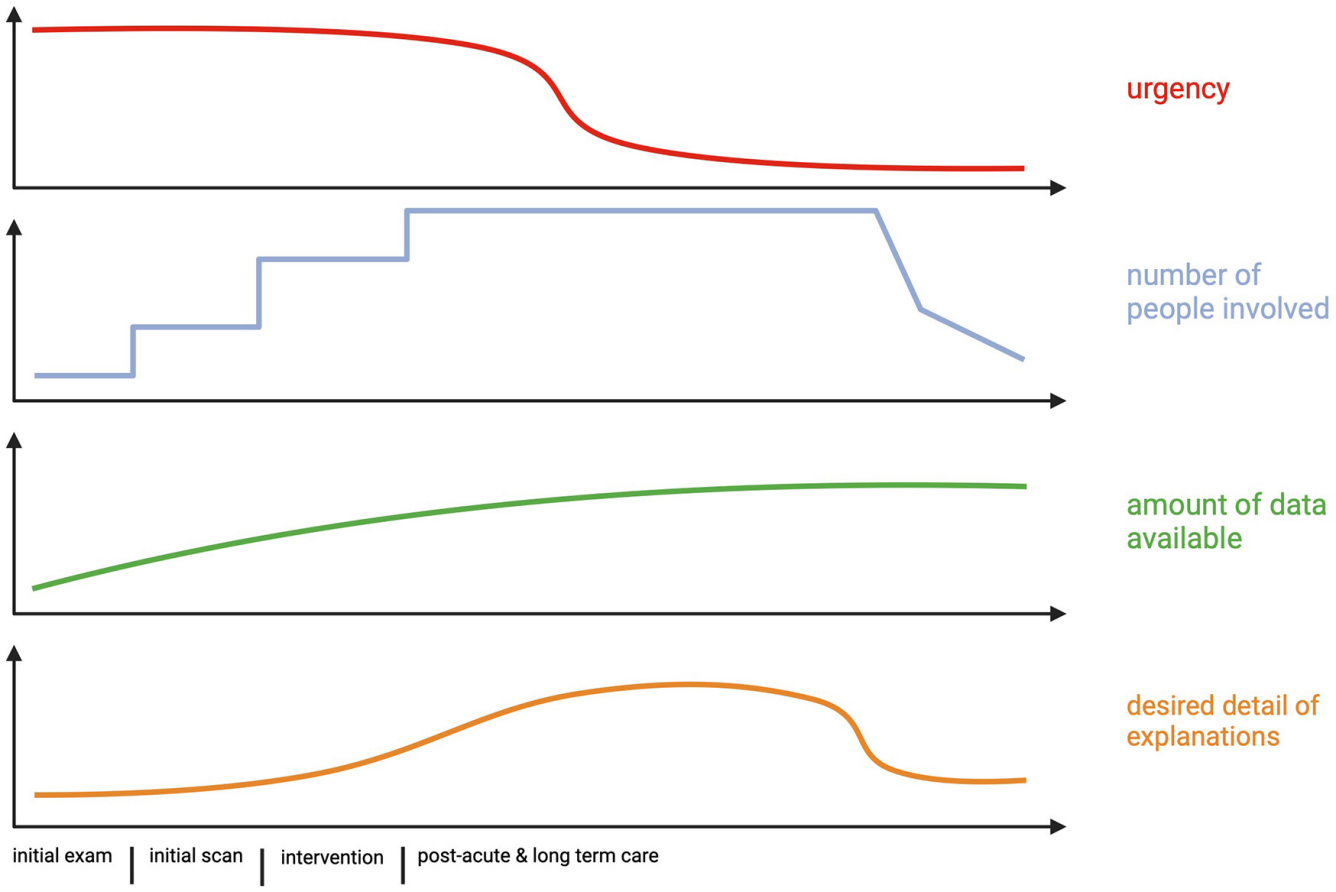
Schematic illustration of different clinical and interpersonal aspects influencing the explanations given by AI, see Section 4. Note that the desired detail of explanations, displayed in the last panel, is an average that may vary between different users.

In this section we provide a simplified example of interactions between an increasing number of professionals in the medical workspace. The use case is divided into four distinct phases, which in reality might overlap and blend into each other. Below we will use this scenario to anchor our discussion of how XAI may transform the medical workspace. Our focus is on different groups of healthcare professionals as users of XAI systems. See Figure 2 for key aspects of this scenario with respect to explanations.

### 3.1 Mr. Smith has a stroke

While having breakfast at 07:20 am, Mr. Smith (73 years old) drops his spoon because of a sudden weakness of the right arm. He is very surprised but can’t articulate properly what happened. His wife is worried and calls an ambulance, and Mr. Smith arrives at the emergency department of a tertiary care center at 08:40 am. Mr. Smith is known to be treated with anticoagulatory medication for atrial fibrillation.

• **Phase 1: Single-person decision.** The resident neurologist is the first physician to see Mr. Smith. He speaks to the patient and carries out a physical examination. He finds that Mr. Smith’s speech is impaired and that he has weakness of the right body side, scoring a total of 10/42 points on the NIH Stroke Scale (NIHSS) (33). The NIHSS is a tool used by healthcare professionals to objectively quantify the impairment caused by a stroke. A high NIHSS score typically indicates a more severe stroke, which may necessitate more aggressive treatment options and often correlates with a poorer prognosis. In the present case, the NIHSS total results from points for severe aphasia (2), partial facial paralysis (2), and no effort against gravity in right arm (3) and leg (3). The neurology resident wants to order imaging of the brain and calls the neuroradiology resident.
• **Phase 2: Two-person interaction.** The neuroradiology resident decides to conduct an emergency CT with CT angiography (CTA). Pre-contrast head CT shows no signs of intracranial hemorrhage and only a small area of weak hypodensity in the left parietal lobe. CTA shows proximal occlusion of the left middle cerebral artery (M1 occlusion). After seeing the large vessel occlusion, the neuroradiology resident adds CT perfusion to the scan, which shows a large area of potentially salvageable brain tissue (penumbra) with a small infarct core. Therefore, the neurology resident now calls the attending neurologist and the interventional neuroradiologist for discussion.
• **Phase 3: Multi-person discussion.** The doctors gather and recap their findings. Based on the information available, they agree that mechanical thrombectomy is the treatment of choice: The patient suffers from a large vessel occlusion that causes significant morbidity. Since there is still salvageable brain tissue, it is expected that Mr. Smith would profit from interventional therapy. The team also discuss thrombolysis, but decide against it due to prior anticoagulatory treatment—although it has been shown that patients treated with direct oral anticoagulants do not necessarily have more bleeding complications, in clinical practice, risks and benefits as well as alternative treatment should be considered (34). Mr. Smith is prepared to go into the catheter lab at 09:15 am (35).
• **Phase 4: Large collective of healthcare workers.** Mr. Smith undergoes thrombectomy, which seems to be successful as he later wakes up in the stroke unit with improved production of speech and motor function of the right body side. In the post-acute phase, many people contribute to optimal care (36). While the neurology resident conducts regular neurological exams, the nurses administer medication and monitor vitals, the physical therapist comes in for early mobilization, the cardiologists are consulted for the stroke work-up, and social services contact Mr. Smith’s wife. They share their findings in a daily report. Eventually, Mr. Smith is discharged and goes back to the care of his general practitioner, who chooses optimal tertiary prevention.

### 3.2 XAI aiding in the stroke scenario

Current AI/XAI systems aiding in stroke diagnosis focus on specific sub-tasks like the detection of intracranial hemorrhage or large vessel occlusion (LVO) in the anterior circulation. Their competence ends with the highlighting of the affected brain region. An optimal XAI system would contribute to the above scenario with more versatility:

• **Phase 1:** If an XAI starts collecting patient information such as NIHSS already during the initial exam, preprocess it and share it with other soon to be involved healthcare providers, the neurology resident could save the time of reporting his findings many times.
• **Phase 2:** While very high performance on LVO detection in the anterior circulation of the brain provides a quick first overview, effectively it does not save any time for the neuroradiologist, as she still needs to check all smaller vessel branches as well as the posterior circulation. Otherwise, a large number of ischemic strokes are missed.
• **Phase 3:** The XAI tool could engage in the multi-person discussion e.g., by highlighting patient information that has not yet been considered or by conducting a real-time search for new evidence-based treatment guidelines.
• **Phase 4:** It could be helpful for the large collective of healthcare workers contributing to Mr. Smith's rehabilitation and prevention to have a joint tool which coordinates appointments. This prevents frustrating trips to an empty patient bed. Additionally, results from daily assessments could be collected, analyzed and visualized in an XAI dashboard, helping to keep track of improvements or deterioration in different aspects of Mr. Smith's health.

To achieve such an optimal XAI system, current technology may be adapted so that it fulfills key requirements, including adaptivity to new data types, hierarchical presentation of information (brief summary first, more information on request) as well as auto-updating of the pre-training medical knowledge base. Further requirements for possible adaptation are found in (20).

## 4 The future of XAI in medicine

### 4.1 Context- and user dependence of explanations

Context- and user-dependence is a key requirement for explanations of AI predictions and decisions. To see why, consider our scenario. When Mr. Smith arrives at the emergency room, his health record may be available in electronic form. A detailed health record helps practitioners to understand current symptoms, for example to gauge the possibility that the right-sided weakness is not new but due to a past stroke. However, large electronic health records also create challenges. For example, the neurologist does not have time to go through vast amounts of data, doing so would create cognitive overload (37, 38). Cognitive overload is avoidable to some extent, because not all data is relevant to the diagnostic situation. Also, even relevant information is not useful if the neurologist does not understand it, cf. Section 2.

Future XAI systems could help with these issues. Once the symptoms, and possibly an initial diagnosis, have been determined and fed into the AI system, the system would filter out and structure currently relevant data. Further in the future, a system may not need manual entry of symptoms and diagnosis, it would automatically generate an initial diagnosis based on symptoms, which are themselves obtained through sensors (cameras, microphones) from the anamnesis and the examination carried out by the physician. The explanation of the diagnosis would be presented to the resident neurologist in an understandable and user-dependent manner, for example by considering the resident’s level of experience and specialization.

What kind of explanation an XAI should generate depends on context; see Figure 1 for key contextual factors. While an explanation of the initial diagnosis is crucial in the emergency room, this information may be less relevant in an ICU, where background information about the patient is assumed to be known (22). Similarly, explanations suitable for research and care contexts may differ considerably (31). In phases 1 and 2 of our scenario, the focus is on care, and a short phrase may be adequate as an explanation, because a longer explanation may delay treatment too much. Explanations in these contexts should also be conservative by only using clinically verified information. For the work-up in phase 4, explanations may be more complete and complex, and it could be highlighted whether information is clinically founded, such that unfounded information can be explored further in research (31).

Building XAI systems that are context- and user dependent creates big challenges. An important design choice is whether different contexts and users are provided to the system as external parameters, or if the system has to infer these parameters. If external parameters are used, parameters have to be set by hand by practitioners each time the context and/or users change, which adds to practitioners’ cognitive load. Also, one may have to create different XAI systems for different parameter settings. This may be feasible if the number of different contexts and users (groups) is limited, but if many fine-grained distinctions between user groups are made, and these distinctions are intersected with fine-grained context distinctions, one ends up with an intractable number of combinations. Alternatively, the XAI system could infer context and user parameters. This would decrease the cognitive burden of practitioners. However, such a system would have to be able to correctly identify users and contexts to provide explanations. In particular, the system would need to reliably distinguish different clinical contexts and situations. But what exactly constitutes a clinical situation? Users and contexts do not carry their requirements on their sleeve, and clinical situations can change quickly. For example, an unconscious patient who was initially thought to have a basilary thrombosis might suddenly become unstable - making other diagnoses, e.g., (covered) aortic rupture, more likely and the situation more urgent. Currently, systems with such abilities do not exist. Creating systems that are fully embedded in a social context is very challenging, as will be argued in more detail below.

It can be questioned whether a customization of explanations for clinical situations and users is necessary. In many clinics, there is an institutional separation between different settings. For example, an XAI system in a post-acute care unit as in phase 4 of the use case will not be confronted with the same kinds of situations as an emergency room in phase 1. This would speak in favor of building different specialized systems for different settings. This points to an important issue for building XAI systems: they are built on the background of existing institutions and need to take these institutions and their inherent logistical challenges into account (39). Building different, walled-off XAI systems for different settings also creates problems. In general, walled-off systems prevent a desirable flow of information between different sites, as witnessed by the benefits of so-called hospital “command centers” (40). Also, walled-off systems may lead to an undesirable reinforcement of institutional structures.

We recommend that the challenge of context- and user dependence should be addressed as follows. First, a design choice must be made, viz., whether the system is supposed to automatically recognize different contexts, such as clinical situations and people. If a system is supposed to do this automatically, it is necessary to have a reliable classification of clinical contexts and people that the system can detect. This classification can be used to build the system, possibly via data labeled by clinical situation, which can also be used to test the system’s capabilities. Such a fully automated system is presumably too challenging to build now. We therefore recommend the more feasible approach of building XAI systems for a particular clinical setting first by introducing a limited set of customizations for different contexts, e.g., different explanations for a limited set of situations and people. The detail and kind of explanation need to be adjustable, depending on the situation and the user. Thus, it will be important to build the system so that it is easy and intuitive to switch between contexts and users.

There are existing and promising lines of research that can be considered to address this challenge. Recent developments in large language models (LLMs) offer promising pathways to address the challenge of tailoring explanations to specific contexts and user groups. One important approach is to augment LLMs with retrieval mechanisms for knowledge-intensive tasks (41, 42). Rather than requiring practitioners to sift through voluminous health records, such retrieval-augmented generation (RAG) methods automatically filter and structure large datasets, retrieving only contextually relevant information. This functionality can reduce cognitive overload by focusing on information that truly matters for the current diagnostic situation, for instance, evidence of past neurological events in a patient’s electronic record.

A key requirement for context- and user-dependent explanations is a sufficiently large “memory” window that allows the AI system to maintain a running record of prior information, thus making explanations consistent over time. Newer transformer-based models and extended-context LLMs enable a more dynamic exchange with users by preserving details of the patient’s condition and the user’s role (e.g., junior resident, attending physician). The ability to adjust the level of detail in explanations based on the user’s experience is enhanced through either fine-tuning or instruction tuning, where the model is specifically optimized to handle domain-specific prompts (43). Furthermore, reinforcement learning from human feedback (RLHF) can be employed to refine these context-aware explanations, ensuring that they remain clinically accurate and appropriately detailed (44).

Technically, this can be realized via a retrieval-augmented multimodal encoder, e.g., Clinical-BERT for notes, a vision transformer for scans, and a tabular net for labs, that fuses modality-specific embeddings into a joint patient representation. Hierarchical XAI then produces saliency overlays on images and Shapley-value rankings on lab features, automatically switching between concise (“emergency”) and detailed (“post-acute”) explanation modes based on context flags.

While these approaches do not yet solve all underlying issues—such as the need to automatically recognize rapidly changing clinical situations—they provide a scalable foundation. Manually specifying parameters for context and user groups remains labor-intensive, but integrating RLHF pipelines allows practitioners to provide feedback on whether an explanation was too long, too technical, or potentially misleading. Over time, systems can learn and adapt to the specific needs of different user groups (e.g., radiologists, neurologists, technicians), reducing the requirement for continuous manual parameter updates.

### 4.2 Human-AI dialogue

So far, we have considered the case of singular explanations: one contextually appropriate explanation is given for an AI output. However, real-life explanations are more dynamical than that. To see why, consider the second phase of our use case, in which an exchange between the radiologist and an imaging system (MRI scan) takes place. The perfusion map shows the infarct core and the penumbra, the areas of which are associated with uncertainty. An XAI system may quantify this uncertainty automatically (45), and visualize it with uncertainty margins that can be thought of as a confidence interval. Further in the future, this process of understanding the result of an imaging procedure may be more dynamical: The radiologist could enter a genuine dialogue with the system to better understand, say, the penumbra image, asking questions and challenging the system’s diagnosis: Why is uncertainty quantified in this way? Is the one-sided weakness really due to a stroke, or rather to a “stroke mimic”, that is, consistent with a stroke but due to a different cause? How can this be determined? and so on.

Is a true human-AI dialogue possible? There are AI systems that show dialogical behavior: One can have “natural” exchanges with large language models (LLMs) such as ChatGPT (46). These models pass medical exams, write grammatically correct and largely coherent answers, modify style based on prompts, and so on. LLMs also face challenges, notably lack of safety (hallucinations), lack of reliance on trusted sources (unreliable citations), and a lack of integration with established knowledge (47). As of now, they also underperform in comparison with more traditional tools in diagnostic contexts (48). The current abilities and limitations of LLMs like ChatGPT are a useful starting point to consider what is needed to create XAI systems for the clinical workspace that can serve as dialogue partners for practitioners like the radiologist.

From a conceptual perspective, we can distinguish the ability to have a genuine dialogue from merely showing dialogical behavior. The former is a capability of humans, while the latter is what LLMs are currently capable of. We propose three properties that can help us to distinguish mere dialogical behavior and genuine dialogue. We use these three properties as a definition of genuine dialogue: They must be satisfied for something to be a genuine dialogue as opposed to mere dialogical behavior. First, having a genuine dialogue requires that the dialogue partners have a representation of the other dialogue participants, and can adapt their contribution to the dialogue to accommodate their dialogue partner, e.g., to make sure one’s contribution is understood, to anticipate what the dialogue partner is likely to know to avoid redundancy, or even to communicate based on empathy with the dialogue partner (49). Currently, LLMs do not have this ability, because they do not form an explicit representation of their dialogue partner. A second requirement for genuine dialogue is the ability to plan and have goals. Usually, one enters a dialogue to achieve a certain communicative goal, to which the dialogue is adapted. For example, while a teacher may provide information freely during normal teaching, they may hold back information during a quiz. Currently, plans are not explicitly represented by LLMs. While they appear to pursue planning in text generation, they do not have the ability to store intermediate results and adapt on this basis (47). A third important aspect of genuine dialogue is that it is self-referential, in that later parts of a dialogue refer to earlier parts, e.g., to get clarification on points raised earlier. This can be observed in LLMs to some extent. Prompts can be used to challenge a model to explain or give a reason for a particular earlier output. Prompting ChatGPT to give reasons for output can improve predictive performance (50). However, it is also known that prompting is fragile, that is, the response to a prompt strongly depends on its exact form (47). Providing an LLM with access to medical records and knowledge databases through retrieval-augmented generation may lead to more personalized treatment (51). It is granted that the three abilities for genuine dialogue may emerge without explicitly building them into the models—LLMs have demonstrated emergent abilities such as constructing implicit world representations from data for predictive purposes (52). But the dialogical abilities are likely to be more stable if they are built into the models.

An XAI system capable of genuine dialogue may fundamentally transform the exchange between the radiologist and the imaging system in the use case. First, if the model has a representation of the dialogue partner, the system can respond in a personalized way to questions, say, about the diffusion image. If the system knows that the radiologist is not very experienced, it responds in a more “didactical” manner, taking into account that junior clinicians may be overly reliant on predictions (53). If the system then detects that its explanation has not been understood from the radiologist’s reaction, it elaborates automatically, or pauses the exchange if it detects fatigue. Second, these considerations would be overridden depending on the goal of the dialogue. If the exchange takes place in an emergency context, the goal of the dialogue changes from didactical to a quick response time, such that the dialogue is more clipped, see the discussion of context. Third, the system would have long-term memory, remembering which students are quicker and which are slower, which colleagues require more detail and which ones just want the gist of the story; explanations that have been given before would be repeated in abbreviated form or skipped altogether.

We recommend that future XAI systems integrate the three properties of genuine dialogue we have identified. The way in which this is implemented is not as important as to have a system with the corresponding functionality. First, the XAI system needs access to information about dialogue partners, and it needs to be able to adapt dialogue behavior to that information. Second, it needs the ability to have plans and goals. Third, it needs the ability to reliably store what it has learned from dialogue partners, and about dialogue partners.

Recent developments in LLMs, already mentioned in the previous section, may offer pathways to address the challenges of creating dialogical AI incrementally. One approach is to augment LLMs with retrieval mechanisms for knowledge-intensive tasks (41, 42). Retrieval mechanisms could be used to tailor the dialogue to specific dialogue partners in a clinical setting if the model has access to information about these dialogue partners. Also, a “memory” window would allow the AI system to maintain a running record of dialogue partners, thus personalizing the exchange. Transformer-based models and extended-context LLMs may enable a more dynamic exchange with users by preserving details of the user’s role (e.g., junior resident, attending physician). Adjusting the level of detail in the explanations based on the user’s properties and abilities could be achieved by fine-tuning or instruction tuning, where the model is optimized with domain-specific prompts (43). Finally, integrating RLHF pipelines could allow practitioners to provide feedback on whether an explanation was adequate for that practitioner, such that the systems can learn and adapt to the specific needs of different user groups. To operationalize genuine dialogue, the XAI pipeline can be wrapped in an LLM-driven QA loop using chain-of-thought prompting and RLHF. On each query, the system emits a one-line rationale (e.g., “perfusion mismatch → penumbra”), logs clinician follow-ups to update a per-user profile, and refines subsequent responses in real time—thereby aligning explanation depth and terminology with individual needs. These suggestions do not address all challenges of dialogical abilities – in particular the requirement of having goals – but provide a first path to address them to some extent.

### 4.3 Social capabilities for XAI

Ultimately, the construction of explanations in the medical workspace relies on non-verbal cues and social facts that are not usually made explicit in conversation. To see why, we turn, again, to our use case. In the third phase, a multi-person decision process takes place. The goal of the process is to reach a decision that maximally benefits the patient, and decision support should be geared towards this goal. This decision process is a collective social endeavor. Collective decision making in medicine may benefit patients in comparison to single decisions (54), but measuring these benefits is challenging (55). Social hierarchy and specialty determine, at least partially, who gets to make the final call; the urgency of a situation may necessitate decision making under considerable uncertainty; and so on. To provide optimal decision support, an XAI system needs social capabilities to “read the medical room.” By social capabilities, we mean the ability to recognize and take into account social aspects of decision making, such as an appropriate interpretation of non-verbal cues and other social facts. The need to add social considerations to obtain an adequate account of medical decision making has been noted in the literature (56). To give an example why this may be the case, imagine that in the stroke scenario, there is a disagreement between different specialties about the best course of action. For example, if Mr. Smith had fewer symptoms and a more distal occlusion, a lower NIHSS would result, and the attending neurologist could argue that the risk of the thrombectomy intervention is not justified in view of limited benefits to the patient. How should such a disagreement be resolved? An XAI system should provide decision support by displaying known options, with pros and cons, and suggestions of how to resolve disagreements. However, collective decision making may also involve purely social abilities. Imagine that a new team member has just started their residency, is intimidated by the environment, and does not speak up about an important finding—it is known that as much as 80% of clinical incidents threatening patient safety may be due to communication errors such as not speaking up (57). An XAI system that registers this omission may push the new resident to speak up. To do this, the system would need social awareness, the ability to understand the social aspects of decision making, and act on this understanding in a socially acceptable manner.

Developing decision support systems with social abilities faces two major challenges. The first challenge is that developing such systems requires the integration of medicine and social science. However, the use of AI in social science has proven to be extremely challenging (58). For example, there have been attempts to develop decision support systems for recruiting with the supposed ability to recognize emotions from videos of applicants in order to determine suitability for a job (59). This kind of application has been viewed skeptically by many AI scholars and has even been dubbed “AI snake oil” (60). The reason is that use of AI for the prediction of social outcomes, such as “job success” or “recidivism within two years,” is not as successful as for more narrow tasks like visual perception or speech recognition. Predicting social outcomes appears to be fundamentally different from a scientific point of view, in particular for outcomes that are further in the future (61). Not only are outcomes less certain, but the social constructs relevant to the outcomes, such as hierarchy, are hard to measure as well (57). Creating systems with the ability to reliably “read a room” lies far in the future.

The second challenge for the development of AI with social abilities is that even if the first challenge could be solved, it is unclear whether adding AI with social abilities to the medical workspace would be an overall benefit because of human feedback. For one, the usefulness of an AI system requires that the intended users of the system actually accept it (62). A system that is socially invasive, e.g., by tracking the physical location of personnel, could be rejected. Whether this is the case is an empirical question. The acceptance of such a system may also go too far in that the humans involved trust the system too much, such that overall performance and safety suffer—this has been observed in the case of simple automated decision making systems in criminal justice (63). The fact that models change, through their presence and their predictions, the underlying distribution to which they are applied, is called performativity (64, 65). Performativity crystallizes the challenge of successfully deploying models in social contexts, including the medical workspace.

We recommend that the second challenge of AI with social capabilities is addressed first. Ultimately, XAI systems should benefit medical professionals, and if medical professionals reject the system, it will not be beneficial. Thus, it must be determined whether and to what extent medical practitioners welcome an XAI system with (moderate) social capabilities, e.g., a system that intervenes in cases where a practitioner does not speak up sufficiently, and where this may have severe consequences. If medical professionals would consider this to be useful, then, in the second step, it needs to be determined to what extent such a system is feasible, integrating technical and social science perspectives. Systems with social capabilities should not be developed if they do not enhance the social system already in place and benefit patients. In the near term, emerging tools from sentiment analysis, affective computing, and Social Signal Processing (SSP) may offer incremental steps toward socially aware XAI. By analyzing vocal tone, facial expressions, response latency, or written communication, a system could detect cues of stress, hesitation, or overconfidence and adjust explanation style accordingly—offering clarification when uncertainty is sensed, or streamlining output when confidence is high. While these methods face domain-adaptation challenges, they could help identify potential communication breakdowns in settings such as clinical team meetings. SSP also opens the door to modeling group dynamics, such as who dominates a discussion or who remains silent (66). Coupled with lightweight trust calibration and multi-agent reinforcement learning, these techniques may one day enable XAI systems to anticipate role conflicts, adapt to different user types, and engage more effectively in social decision-making contexts (67). As stressed above, these directions require caution to avoid the pitfalls of “AI snake oil.”

Any move toward greater social awareness should be accompanied by user-centered design principles. Approaches such as participatory design and iterative prototyping, where clinicians co-create features and validate their usability, can help prevent systems from becoming overly intrusive or undermining professional trust (68). During pilot projects, the AI might, for instance, nudge a junior resident to articulate a critical observation only when the situation carries a high risk for patient safety, thus striking a balance between beneficial intervention and excessive monitoring. Ultimately, a measured approach, built through continuous feedback from healthcare professionals and refined by limited deployments, could allow these frameworks to evolve organically without disrupting the delicate social fabric of medical teams. If accepted and proven useful, further integration of technical and social science methods may enable AI to “read the medical room” more reliably, complementing clinicians’ expertise in collective decision-making and thereby fostering safer and more equitable patient care.

## 5 Conclusion

We examined the integration of XAI into the future clinical routine, based on a stroke scenario, a simplified, full treatment cycle. We identified three dimensions along which XAI should be adapted to be fully integrated into this routine. First, we argued that explanations should be context- and user-dependent. This creates the challenge of either creating multiple systems for different contexts and users, or of providing XAI with the ability to autonomously differentiate contexts and users. Second, we analyzed the prospect of creating dialogical explanations against the backdrop of LLMs like ChatGPT. We proposed three properties that separate these models from genuine dialogue: representation of the dialogue partner, planning, and goal dependence, and the ability to draw on previous parts of the dialogue. We found that these properties are (mostly) absent from current models. Third, we argued that future XAI systems may need social abilities. We found that this will necessitate the integration of medicine and methods from social science; the latter creates unique hurdles for AI in general.

How should XAI be operationalized in clinical practice on the basis of the above discussion? First, awareness of the issues and limitations of current AI systems should be created; this would demonstrate the need for (current and future) XAI. This milestone has been reached to some extent; however, there is still a lack of awareness of the practical value of XAI among some medical practitioners (14). Second, clinical evaluation frameworks for XAI have to be created and run. Such frameworks could be similar to Quantus (17), but should focus more on clinical scenarios. The purpose of using these frameworks should be to produce evidence on the clinical impact of XAI on AI-based tools in clinics. Third, medtech companies should be involved in choosing and refining XAI technologies so that they can be certified, deployed, and improved.
